# Metabolic Reprogramming by Ribitol Expands the Therapeutic Window of BETi JQ1 against Breast Cancer

**DOI:** 10.3390/cancers15174356

**Published:** 2023-09-01

**Authors:** Ravi Doddapaneni, Jason D. Tucker, Pei J. Lu, Qi L. Lu

**Affiliations:** McColl-Lockwood Laboratory for Muscular Dystrophy Research, Atrium Health Musculoskeletal Institute, Wake Forest University School of Medicine, 1000 Blythe Blvd., Charlotte, NC 28231, USA

**Keywords:** breast cancer, ribitol, JQ1, metabolic reprogramming, cancer therapy

## Abstract

**Simple Summary:**

Safety and pre-existing or acquired resistance to treatments limit the clinical benefit of most drugs against breast cancer. Great effort has been made to mitigate the limitations of combined drug treatment. We here explored the effect of the combination of ribitol, a sugar metabolite, with the bromodomain inhibitor JQ1 on breast cancer cells. Our results show that ribitol selectively synergizes with JQ1 to inhibit cell growth and migration and enhance cell death by apoptosis. This effect is cell type specific and associated with an alteration in glycolysis and the expression of the genes involved in cell survival and death. Ribitol with limited side effect is highly desirable for clinic applications, and a reduced dosage of JQ1 would mitigate its toxicity. The results also emphasize the importance of selective applications of targeting metabolism for cancer treatment.

**Abstract:**

Many cancer patients still lack effective treatments, and pre-existing or acquired resistance limits the clinical benefit of even the most advanced medicines. Recently, much attention has been given to the role of metabolism in cancer, expanding from the Warburg effect to highlight unique patterns that, in turn, may improve diagnostic and therapeutic approaches. Our recent metabolomics study revealed that ribitol can alter glycolysis in breast cancer cells. In the current study, we investigate the combinatorial effects of ribitol with several other anticancer drugs (chrysin, lonidamine, GSK2837808A, CB-839, JQ1, and shikonin) in various breast cancer cells (MDA-MB-231, MCF-7, and T-47D). The combination of ribitol with JQ1 synergistically inhibited the proliferation and migration of breast cancer cells cell-type dependently, only observed in the triple-negative MDA-MB-231 breast cancer cells. This synergy is associated with the differential effects of the 2 compounds on expression of the genes involved in cell survival and death, specifically downregulation in c-Myc and other anti-apoptotic proteins (Bcl-2, Bcl-xL, Mcl-1), but upregulation in p53 and cytochrome C levels. Glycolysis is differentially altered, with significant downregulation of glucose-6-phosphate and lactate by ribitol and JQ1, respectively. The overall effect of the combined treatment on metabolism and apoptosis-related genes results in significant synergy in the inhibition of cell growth and induction of apoptosis. Given the fact that ribitol is a metabolite with limited side effects, a combined therapy is highly desirable with relative ease to apply in the clinic for treating an appropriate cancer population. Our results also emphasize that, similar to traditional drug development, the therapeutic potential of targeting metabolism for cancer treatment may only be achieved in combination with other drugs and requires the identification of a specific cancer population. The desire to apply metabolomic intervention to a large scope of cancer types may be one of the reasons identification of this class of drugs in a clinical trial setting has been delayed.

## 1. Introduction

Breast cancer is the most common cancer and one of the leading causes of cancer-related death [1]. The World Health Organization estimates that breast cancer accounted for 12% of all new cancers diagnosed worldwide in 2021 [2]. Several decades of intensive studies have led to impressive progress in early diagnosis and treatment. New drugs targeting many different pathways have been developed and tested in clinical trials. More recent developments targeting epidermal growth factor receptor 2 (HER2) and immune checkpoint molecules have demonstrated highly encouraging efficacy in a subset of patients. However, most breast cancers remain uncurable when tumors are non-excisable. Therefore, therapeutic strategies must be further developed, including ways to improve existing treatment regimes. 

Recently, our group reported that the pentose alcohol ribitol has the capacity to enhance the production of CDP-ribitol in muscle tissues and restore the expression of matriglycan in a dystroglycanopathy mouse model with FKRP mutations [3]. This led to a significant improvement in muscle pathology and function. Further, our results demonstrate for the first time that ribitol and CDP-ribitol were able to enhance matriglycan in breast cancer cells [4]. This effect in glycosylation was not associated with an alteration in FKRP or LARGE expression, therefore suggesting a new pathway of metabolite-mediated modulation of matriglycan. Unexpectedly, the enhancement in matriglycan does not inhibit the growth of breast cancer cells of luminal type positive for ER and PR (MCF-7 and T-47D). This result contrasts with early reports that enhanced matriglycan expression is associated with inhibition of cancer growth and invasion [5,6,7]. Our follow-up study to understand the metabolic effect of ribitol by metabolomics revealed that ribitol-induced enhancement in matriglycan is associated with an alteration in glycolysis in MCF-7 cells [8]. Considering that breast cancers are heterogeneous in cell types with variation in metabolic pathways, the effect of ribitol treatment on cell growth may vary cell-type dependently. It is therefore desirable to investigate the effect of ribitol on other cancer cell types to explore its therapeutic potential, including its use in combination with existing chemotherapeutics, considering that ribitol is extremely safe to use, as demonstrated in both animal and clinical trials in muscular dystrophy patients. In this study, we investigated the response of different breast cancer cells (MDA-MB-231, MCF-7, and T-47D) to ribitol and other selected drugs that target crucial metabolic enzymes of central carbon metabolism such as chrysin (succinate dehydrogenase), CB-839 (glutaminase), GSK2837808A (lactate dehydrogenase), lonidamine (hexokinase), JQ1 (lactate dehydrogenase), and shikonin (pyruvate kinase) and the effect of their combination on cancer cell growth and invasion as well as mechanism of action. 

An emerging hallmark of cancer is the altered metabolism relative to normal cells [9,10]. Most cancer cells rely on glycolysis to generate ATP, even when oxygen is available [11,12,13,14]. Earlier studies also reported that glucose metabolism can be the sole source of energy for cancer cells [15]. This hallmark can be reflected in the changes in levels of many metabolites, for example, increased levels of lactate in cancer cells [16,17,18]. Nevertheless, to date, only a few studies have explored the impact of drugs on metabolic pathways in breast cancer. This led to the consideration that inhibition of glycolysis could be an effective means to inhibit or even kill cancer cells. Though efforts to target glucose uptake or lactate production have found some success in preclinical models [19,20], clinical success has been limited [21]. Studies further demonstrated that changes in metabolism are more complex in cancer cells, which have an extremely high ability to utilize alternative metabolic pathways to survive and grow in response to changing growth environments [22,23]. It is now generally agreed that targeting metabolic pathways for cancer treatment would probably have a significant effect only when used in combination with other targeted treatments. Taking advantage of metabolomics, a rapidly developing suite of approaches that permit comprehensive analysis of metabolites, we also proceeded to assess the differential effects of ribitol and the lactate dehydrogenase inhibitor JQ1 on various metabolic pathways.

Our findings suggest that ribitol with JQ1 administration affects a wide range of metabolic pathways cell-type dependently, and the combination of ribitol and JQ1 inhibits breast cancer cell proliferation and migration. This effect is attributed to the synergy of both ribitol and JQ1 on cancer cell metabolism and an alteration in gene expression related to cell death and survival. Our results provide insight into appropriate strategies for combination therapy targeting cancer metabolism in different types of cancer cells. 

## 2. Materials and Methods

### 2.1. Cell Lines and Culture

The human breast cancer cell lines MCF-7 (ATCC-HTB-22), T-47D (ATCC-CRL2865), and MDA-MB-231 (ATCC-HTB-26) were purchased from ATCC (Manassas, VA, USA). MCF-7 and T-47D were cultured in DMEM-GlutaMAX (Life Technologies, Carlsbad, CA, USA) plus 10% fetal bovine serum (FBS 10082-147, R&D Systems) and 10µg/mL insulin (I5500 Sigma-Aldrich, St. Louis, MO, USA). MDA-MB-231 were grown in DMEM-GlutaMAX + 10% FBS at 37 °C in a 10% CO_2_ incubator. The culture media were obtained from Gibco by Life Technologies.

### 2.2. Measurement of Glucose and Lactate Levels

In order to determine glucose and lactate levels, cells (4 × 10^4^ per well) were cultured for 72 h in experimental conditions and processed per the manufacturer’s instructions. Briefly, a handheld glucometer utilizing glucose oxidase strips (Bayer) was calibrated and tested on solutions of known glucose concentration to ensure accuracy, followed by measuring experimental replicates for glucose concentrations. The Lactate Assay Kit (MAK064 Sigma Aldrich) was used to determine the concentrations of lactate in cancer cell cultures that were treated with the indicated nutrients. In this assay, lactate concentration is determined by an enzymatic assay, which results in a colorimetric (570 nm)/fluorometric (λex = 535 nm/λem = 587 nm) product proportional to the lactate present. Plates were read with a Biotek Synergy plate reader in colorimetric or fluorescent mode as each kit required, and data were exported to Excel for analysis.

### 2.3. Compound Screening

For primary screening, cells were pretreated with or without 5 mM ribitol for 72 h in plates. Then, 8000 cells per well were seeded in a 96-well plate. Along with ribitol, we screened a panel of drugs, including chrysin (Sigma), CB-839 (Selleckchem, Houston, TX, USA), GSK2837808A (Sigma), lonidamine (Selleckchem), JQ1 (Selleckchem), and shikonin (Selleckchem), in escalating doses. Cancer cell lines were treated with single agents and combinations of ribitol with other compounds at equimolar concentrations of 10–0.16 µM. Cell viability was determined after 72 h using CellTiter-Glo reagents (Promega, Madison, WI), following the manufacturer’s instructions. Viability was measured with a Biotek multi-label reader (Perkin Elmer, Waltham, MA, USA) as a percentage of response relative to both cells treated with DMSO alone (0% response). 

### 2.4. Cell Viability Assay and Synergy Software

In the secondary screen, as JQ1 was identified as one of the top drugs involved in synergistic combinations with ribitol, it was chosen as the anchor drug. Cell lines were treated for 72 h with ribitol in single applications and in combinations with JQ1 in all possible combinations of doses. Cell viability was determined after 72 h using Cell Titer-Glo (Promega, Madison, WI, USA), following the manufacturer’s instructions. These results were analyzed using Combenefit software (version 2.021). We configured a 96-well microplate assay compatible with the Combenefit dual-drug interaction software; cell concentration data, read spectrophotometrically, were submitted to rigorous statistical analysis for synergistic or antagonistic interactions, calculated according to the Loewe additivity model, which is part of the Combenefit package. Combenefit software was used to perform synergism determination. The Combenefit software package calculates and displays the synergism-antagonism distributions and computes a variety of metrics from the distributions. The dose–response curve for each of the chemotherapeutic drugs is computed by the software from all biologic replicates of that drug combination.

### 2.5. Wound Healing Assay

In vitro migration (scratch) assay was carried out in MDA-MB-231 cells. The cells were seeded in 12-well culture plates in complete media and incubated overnight. After reaching confluence, a uniform scratch was made in the center of the well using a micropipette tip, and a baseline image was taken of the entire scratch width. Cells were then treated with half of the EC_50_ concentrations determined for JQ1. Cells were incubated for 48 h in a MuviCyte (PerkinElmer, Waltham, MA, USA) Live-Cell imaging system attached to an incubator at 37 °C in 5% CO_2_. Microphotographs were captured at 2 h intervals via the MuviCyte Live-Cell imaging software version 2.0.26 (PerkinElmer) and analyzed.

### 2.6. Immunocytochemistry (ICC)

Cell cultures for ICC were washed with PBS before fixation with ice-cold methanol for 10 min. Residual methanol was removed by washing with PBS and air-drying. Cells were rehydrated prior to staining procedures with PBS and blocked with 6% bovine serum albumin (BSA) and 2% normal goat serum (NGS) in PBS for 30 min. Primary anti-cytochrome C antibodies (D18C7, Cell Signaling, Danvers, MA, USA) and mouse anti β-actin antibodies (AC-74, Sigma-Aldrich, St. Louis, MO, USA) in 1% BSA at 1:600 dilution were incubated for 4 h at RT or overnight at 4 °C. Samples were washed three times for 10 min with PBS and finally incubated with Alexa Fluor 488-conjugated goat anti-mouse IgG or Alexa Fluor 594-conjugated goat anti-Rabbit IgG secondary antibodies (Life Technologies, Carlsbad, CA, USA) at 1:600 dilution. Samples stained without primary antibody were used as secondary controls.

For propidium iodide (PI)/Hoechst 3032 staining, cells plated at 4 × 10^4^ per well after 72 h of culture in experimental conditions were processed by addition of propidium iodide (P3566 Invitrogen, Waltham, MA, USA) at 20 µg/mL and Hoechst 33342 (H3570 Invitrogen) at 5 µg/mL. After the addition of dyes, the cells were incubated at room temperature in the dark for 10 min, and fluorescent microscopy images were captured in triplicate or more per treatment on an Olympus IX71 inverted microscope. 

### 2.7. RealTime-Glo™ Annexin V Apoptosis Assay 

The purpose of this experiment was to determine the apoptotic activities of combination therapy in comparison to monotherapy. Briefly, MDA-MB-231 cells were treated for 48 h with the EC_50_ of JQ1 in combination with ribitol. In the RealTime-Glo™ Annexin V Apoptosis and Necrosis assay, time- and dose-dependent increases in luminescence are produced—the annexin V fusion protein binds to exposed phosphatidylserine (PS), which leads to temporal increases in fluorescence due to secondary necrosis via loss of membrane integrity. In this experiment, we have analyzed the response of the breast cancer cells treated with ribitol (5 mM) and JQ1 (0.5 µM and 1 µM) over 48 h. Readings were measured on an Envision multi-label reader (Perkin Elmer, Waltham, MA, USA) as a percentage of response relative to cells treated with DMSO alone (0% response).

### 2.8. Western Blot

The level of apoptotic proteins was assessed by subjecting 60 µg of total cell lysates to immunoblot analysis. Cells were lysed in Triton lysis buffer containing 1% Triton X-100, 50 mM Tris pH 8, 150 mM NaCl, 1 mM EDTA, and 1× protease inhibitor cocktail (Sigma). After clarification of the lysates by centrifugation at 13,000 rpm for 10 min at 40C, protein concentration of the lysates was measured using the Bradford method (BioRad, Hercules, CA, USA). Samples were then electrophoretically separated on a 4–15% Criterion Tris-HCI 18-well gel (3450028, Bio-Rad Laboratories) and transferred onto a supported nitrocellulose membrane. Immunoblots were probed with primary antibodies c-Myc (SC-40, Santa Cruz Biotechnology, Santa Cruz, CA, USA), p53 (SC-126, Santa Cruz), Bcl-2 (SC-509, Santa Cruz), Bcl-xL (AB32370, Abcam, Cambridge, UK), and Mcl-1 (SC-12756, Santa Cruz) at 1:1000 dilution in 5% nonfat dry milk/1 × TBS-0.05% Tween. Rabbit polyclonal antibody to actin (A2066 Sigma) was used at 1:3000 dilution in 5% nonfat dry milk/1 × TBS-0.05% Tween as loading control. The blots were incubated with primary antibody overnight at 40 °C. After washing, membranes were subsequently incubated with secondary antibodies of HRP-conjugated goat anti-mouse IgG (1:3000) or goat anti-rabbit IgG (1:3000) in their blocking buffer for 1 h 30 min. Bands were detected using the ECL Detection Kit NEL 104001EA (PerkinElmer) on blue basic autoradiography film (USA Scientific, Ocala, FL, USA).

### 2.9. Metabolomics

Cell pellets from 72 h cultures with ribitol, JQ1, or an untreated control were collected via scraping and centrifugation, after a PBS wash, and submitted to the West Coast Metabolomics Center (WCMC) core for metabolomic analysis. Primary metabolomic analysis was performed with GCTOF-C in a resuspension volume of 100 µL with an injection volume of 0.5 µL. A total of 167 metabolites were identified after data processing, with study data normalized to the average mTIC for the respective sample type. Key metabolites (47) were identified and fold change (Log2) and statistical significance were calculated between experimental groups by *t*-test.

### 2.10. Statistical Analysis 

All observations in this study were analyzed in triplicate or more, and each experiment was repeated three times. Dose–response data were analyzed by ANOVA followed by a Tukey post hoc comparison of all the means to determine significance. Values represent the mean ± SEM of three independent experiments. To compare two groups, student’s *t* test was used, and *p* < 0.05 was considered statistically significant.

## 3. Results

### 3.1. Differential Effect of Ribitol on Glucose and Lactate Levels in Breast Cancer Cells 

**Figure 1:**
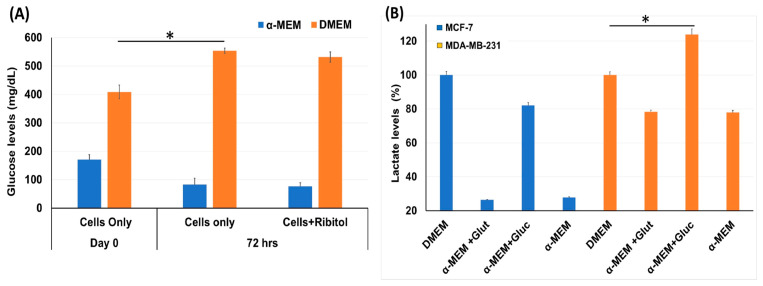
Measurement of glycolysis pathway molecules glucose and lactate level. (**A**) Glucose levels in MDA-MB-231 cells incubated with ribitol for 72 h in both DMEM and α-MEM media. (**B**) Lactate levels in MCF-7 and MDA-MB-231 cells cultured in DMEM and α-MEM media. Taken DMEM as a control to compare the lactate levels for analysis of different groups and read at OD570. Data are calculated from triplicate experiments. * Indicates a significant (* *p* < 0.05) difference compared with control. All data are mean ± SEM.

Considering that breast cancers are heterogeneous in cell types with variation in metabolic pathways, the effect of ribitol treatment may vary cell-type dependently. We therefore first assessed the effect of ribitol on levels of glucose and lactate, important indicators for the status of glycolysis in MDA-MB-231 cells (Figure 1). We found a significant (*p* < 0.05) difference in levels of glucose between control cells at day 0 and 72 h (Figure 1A) when cultured in DMEM. Consistent with early reports, we observed a significant (*p* < 0.05) increase in lactate levels between control cells (DMEM) and cells cultured in the presence of glucose in basal-like MDA-MB-231 cells compared to luminal-like MCF-7 cells (Figure 1B). In particular, the lactate level was relatively elevated in MDA-MB-231 cells at both culture conditions with high and low glucose levels, a clear indication that MDA-MB-231 cells had greater glycolytic potential than MCF-7 cells. The observed differences in the levels of the two metabolites indicate variations in the glycolysis pathway between MCF-7 and MDA-MB-231 cells.

### 3.2. Ribitol Selectively Synergize with JQ1 to Enhance Cytotoxicity in Breast Cancer Cells

**Figure 2:**
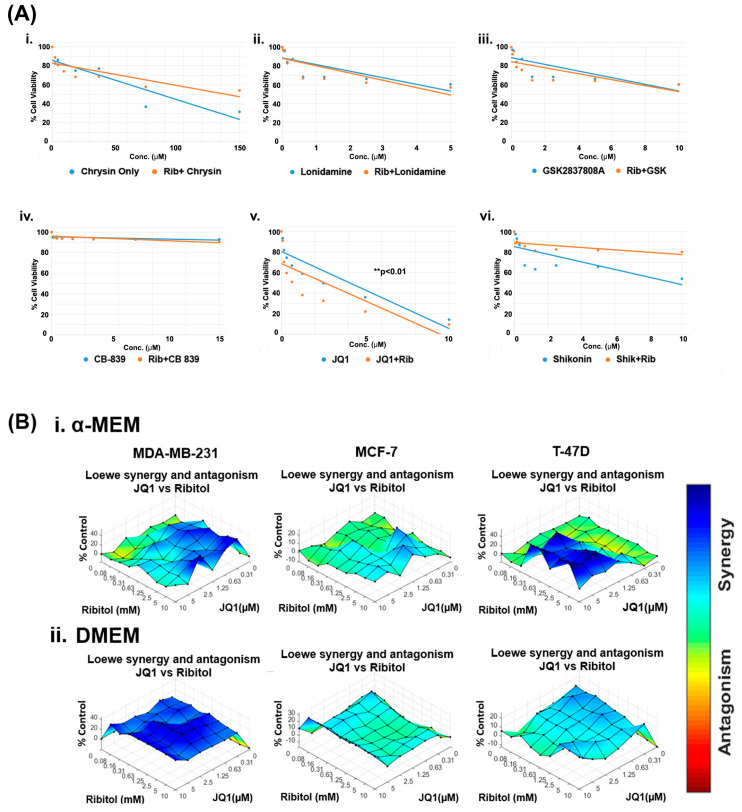
Ribitol treatment enhances the efficacy of JQ1. (**A**) Dose-response curve of MDA-MB-231 cells (i) Chrysin, (ii) Lonidamine, (iii) GSK2837808A, (iv) CB-839, (v) JQ1, and (vi) Shikonin with and without ribitol treatment measured using ATP detection (CellTiter-Glo assay). (**B**) Synergistic effect of ribitol and JQ1 on MDA-MB-231, MCF-7 and T-47D cells cultured under both α-MEM and DMEM media. Synergy between ribitol and JQ1 is analyzed by surface matrix plots. Data are expressed as the mean ± SEM from three independent experiments. ** *p* < 0.01 indicate significant difference compared to the untreated control.

Currently, the overwhelming treatment strategy is to apply a combination of drugs targeting different pathways for enhanced efficacy with minimized toxicity. This is especially the case when considering altering metabolism as a cancer treatment. The differential effect of ribitol in glycolysis between MCF-7 and MDA-MB-231 breast cancers prompted us to hypothesize that the therapeutic potential of ribitol could be explored by combinatorial treatment with other cancer drugs that target various metabolic pathways such as glycolysis, the TCA cycle, and glutaminolysis. We therefore screened a panel of drugs, including chrysin (succinate dehydrogenase), CB-839 (glutaminase), GSK2837808A (lactate dehydrogenase), lonidamine (hexokinase), JQ1 (lactate dehydrogenase), and shikonin (pyruvate kinase), in escalating doses in combination with 5 mM ribitol in MDA-MB-231 breast cancer cells (Figure 2). Chrysin and shikonin did not show any synergistic behavior with ribitol in the cells. The combination of ribitol with lonidamine and lonidamine alone had similar cell viability, indicating no impact of ribitol on lonidamine. Similar findings were observed with ribitol on the GSK2837808A. The glutaminase inhibitor CB-839 had minimal effect on the viability of these cells. As shown in Figure 2(Av), another lactate dehydrogenase inhibitor, JQ1, alone decreased the viability of cells, and JQ1 in combination with ribitol enhanced such an effect significantly (** *p* < 0.01) compared to the untreated control. Therefore, we focused our further efforts on the therapeutic potential of ribitol and JQ1 for the treatment of various types of breast cancer.

To determine the dose range and cell specificity for synergy by the combined treatment of JQ1 and ribitol, we examined three breast cancer cells of different phenotypes, MDA-MB-231, MCF-7, and T-47D cells, side-by-side with ribitol and JQ1 at varying concentrations. The cells were cultured under both DMEM (high glucose) and α-MEM (low glucose) media to assess the potential effect of nutritional components in the culture media. The half maximal effective concentration (EC_50_) values for JQ1 in cells cultured under α-MEM media were 2.6 mM, 10.7 mM, and 23.8 mM for MDA-MB-231, MCF-7, and T-47D cells, respectively (Supplementary Figure S1A). JQ1 and ribitol inhibited proliferation of the different types of breast cancer cells (MCF-7, T-47D, and MDA-MB-231 cells) dose dependently (Supplementary Figure S1B). As shown in Figure 2B, synergistic effects started to occur at concentrations of ribitol and JQ1 as low as 0.16 mM and 0.31 µM, respectively, in the α-MEM medium for MDA-MB-231 cells. Synergy becomes strongest with an increase in ribitol concentration up to 10 mM, with JQ1 at 2.5 µM. A further increase in JQ1 concentration achieved less synergy, as shown by synergistic surface matrix plots in Figure 2(Bi), which were generated by Combenefit software (version 2.021) data analysis. As shown in Figure 2(Bii), a synergistic effect was observed significantly (*p* < 0.001) with a wider range of both JQ1 and ribitol concentrations in cells cultured under DMEM media than under α-MEM, suggesting the importance of nutrient composition on the response of cancer cells to chemodrug and ribitol. Under the same combinatorial treatments of the two agents, synergy was only detected at the highest concentration of JQ1 with ribitol in the T-47D cells incubated in the α-MEM, but not in the DMEM. In contrast to MDA-MB-231 cells, there was no synergy between ribitol and JQ1 in MCF-7 cells. Taken together, these results suggest that synergy between ribitol and JQ1 on inhibition of cancer cell growth is determined by both the nutritional environment and the phenotype of cancer cells. 

### 3.3. The Combined Treatment of Ribitol and JQ1 Restricts MDA-MB-231 Cell Migration 

**Figure 3:**
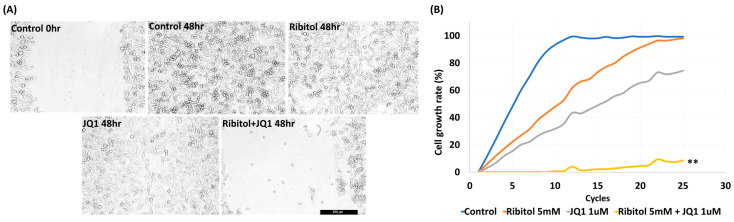
Synergistic effect of ribitol and JQ1 on MDA-MB-231 cell migration and proliferation. (**A**) Representative images of migration assay after the cells were treated for 48 h. Control 0 h, image was taken immediately after the scratch in the control cells; control 48 h, image was taken after 48 h of scratch in the control cells. Shown are the representative images at 100× magnification (scale bar = 250 µm); (**B**) growth curve analysis of the cells over 48 h of ribitol, JQ1, and combinatorial treatment. Untreated cells were used as a control. All data are mean ± SEM. *p* < 0.05 considered significant. ** *p* < 0.01 compared with untreated control.

Our earlier work showed that ribitol alone did not affect the matriglycan expression and growth of MDA-MB-231 cells significantly. However, the effect of ribitol on cell migration has not been evaluated. We therefore performed a wound-healing assay to determine the effect of ribitol alone and in combination with JQ1 on the migration of the cells. Ribitol (5 mM) or JQ1 (1 μM) showing high synergy as described above were applied to the cells cultured in the DMEM medium. Once the scratch was made across the center of the wells, a live cell imaging system was used to record the movement of the cells migrating towards the gap over a period of 48 h (Figure 3). Moreover, we recorded a 48 h live video of the migration of breast cancer cells after treatment by the MuviCyte Live-Cell imaging system (see Supplementary Video S1).

The results showed that MDA-MB-231 cells exhibited a >95% (99.5 ± 1.25%) bridging of the acellular gap over the period, whereas the cells treated with ribitol (5 mM) had an 88.7 ± 3.12% closure (Figure 3A), and the cells treated with JQ1 (1 μM) alone exhibited a 68.5 ± 1.57% (Figure 3A ** *p* < 0.01 wrt control) bridging at this concentration (Figure 3A). However, cells treated with the combination of ribitol and JQ1 exhibited only 12.5 ± 2.12% bridging, which represented a decrease in migratory activity when compared to controls as well as to the single-agent treatment of ribitol and JQ1 (Figure 3A). Furthermore, the combination treatment also decreased overall cell numbers over the 2-day period, corroborating the finding of a synergistic effect on cellular viability (Figure 3B). The combined cytotoxicity effect was significant (*p* < 0.01) when compared to either drug alone. The results further support the notion that a metabolite of ribitol at a non-cytotoxic concentration could promote an anticancer effect from JQ1, which is highly desirable for an initial clinical trial as a combinatorial treatment for a specific breast cancer population.

### 3.4. Ribitol Enhanced JQ1 Toxicity Is Mediated with Cell Death by Apoptosis

**Figure 4:**
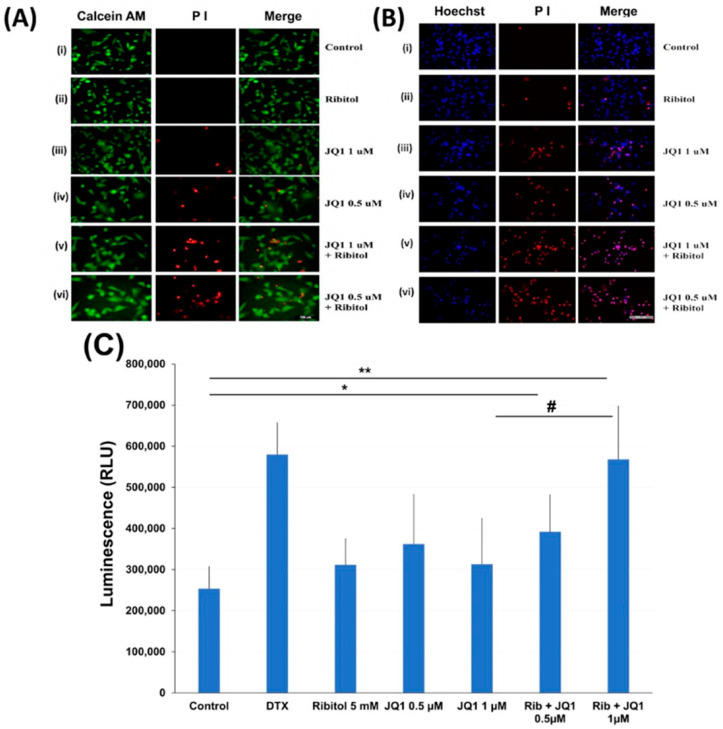
Apoptosis of MDA-MB-231 cells after treatment with ribitol and JQ1. (**A**) Live/dead assay of breast cancer cells incubated with (**i**). Control; (**ii**) ribitol 5 mM; (**iii**) JQ1 1 µM; (**iv**) JQ1 0.5 µM; (**v**) ribitol 5 mM and JQ1 1 µM; (**vi**) ribitol 5 mM and JQ1 0.5 µM after 72 h of incubation; (**B**) Representative images of propidium iodide (PI)/Hoechst 33342 staining of the cells treated with (**i**). Control; (**ii**) ribitol 5 mM; (**iii**) JQ1 1 µM; (**iv**) JQ1 0.5 µM; (**v**) ribitol 5 mM and JQ1 1 µM; (**vi**) ribitol 5 mM and JQ1 0.5 µM after 72 h of incubation. Shown are the representative images at 200× magnification (scale bar = 100 µm); (**C**) the apoptosis measurements are expressed as total luminescence and measurements of luminescence signals as relative luminescence units (RLU). Breast cancer cells were exposed to ribitol and JQ1 in the presence of RealTime-Glo^TM^ Annexin V Apoptosis assay reagent. The cells were treated for 72 h. Docetaxel (DTX) was used as positive control to induce apoptosis. Each value represents the average of the independent experiments with triplicate determinations. * Significant compared to control cells (* *p* < 0.05, ** *p* < 0.01). # *p* < 0.05 indicates a significant difference between the JQ1 1 µm treated cells and combination treated cells. All data are mean ± SEM.

To understand the mechanisms for ribitol-enhanced JQ1 toxicity, we first examined the effect of ribitol and JQ1 with a live/dead assay on the MDA-MB-231 cells treated with ribitol at 5 mM and JQ1 at 1 mM and 0.5 mM concentrations (Figure 4A). Green fluorescence of calcein AM labeling showed most of the cells were alive, with only a few dead cells (stained by the dead cell marker PI) in both control and ribitol-treated samples. This result confirms that ribitol itself has a very limited effect on the viability of the cells. JQ1 at both concentrations decreased the cell viability significantly, as shown by the PI staining (Figure 4(Aiii,Aiv)). As expected, more dead cells were detected in the cells treated with both ribitol and JQ1 (Figure 4(Av,Avi)). Background fluorescence levels are inherently low with this assay because the dyes are virtually non-fluorescent before interacting with cells. 

Cell death was also assessed with PI/Hoechst 33342 staining and examined by fluorescent microscopy. Representative images of cells stained with Hoechst/PI are shown in Figure 4B. We noticed some important observations after cancer cells were treated with ribitol and JQ1. First, the total number of cells (Hoechst staining) is reasonably less and is markedly less than the number of dead cells (PI staining) in combination of ribitol and JQ1 treated cells compared to single agent treatment and control cells. Second, since only cells labeled with both Hoechst and PI are counted as dead (purple cells in the merged image), cells were noticed in combination treatment in a greater proportion than in single treatment (Figure 4(Biii,Biv). These results suggest that the combination of ribitol and JQ1 induces cell death in these cancer cells.

The nature of the cell death was therefore analyzed by the real-time Glo annexin V apoptosis luminescence method. MDA-MB-231 cells were treated with JQ1 with and without ribitol at the indicated concentrations for 72 h and the luminescence was then measured (Figure 4C). As illustrated in Figure 4C, ribitol alone increased apoptotic cells (without statistical significance) when compared to the control cells. However, when compared to control cells, combined treatment of ribitol and JQ1 (0.5 µm and 1 µM) increased the apoptotic cells significantly (*p* < 0.05 and *p* < 0.01), showing the highest luminescence with intensity equal to positive control cells treated with DTX. These results support the notion that combined treatment with ribitol and JQ1 induces cell death by apoptosis.

**Figure 5:**
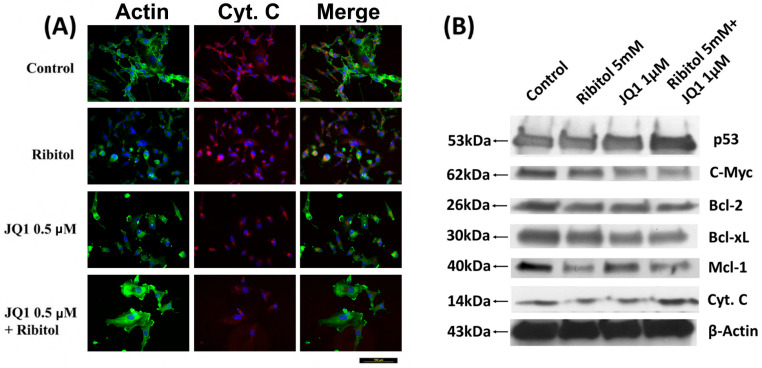
Immunocytochemical analysis of cytochrome C and Western blot analysis of apoptotic proteins. (**A**) Representative images of immunofluorescence analysis of cytochrome C staining of control, ribitol, JQ1, and ribitol+JQ1. After treatment of indicated doses of agents, MDA-MB-231 cells were incubated for 72 h, stained with anti-cytochrome C antibody, and photographed under fluorescent microscopy. Original magnification: 200× (scale bar = 100 µm). (**B**) Representative images of various pro/anti-apoptotic proteins, p53, c-Myc, Bcl-2, Bcl-xL, Mcl-1, and cytochrome C, by Western blot of MDA-MB-231 cells after treatment with ribitol and JQ1. Original western blots are presented in File S1.

Since cytochrome C (Cyt C) release is a key event that mediates the apoptotic pathway, its levels and distribution pattern were also examined (Figure 5). Fluorescence staining with the Ab to Cyt C demonstrated mainly punctuate signals with one large patch of perinuclear signal in most control cells and the cells treated with ribitol and JQ1 alone (Figure 5A). This pattern is consistent with the perinuclear Golgi localization of Cyt C in the cultured cells. However, in the cells with combined treatment, the signal for Cyt C appeared largely as diffused cytoplasmic staining without the concentrated Golgi localization. This result further supports the notion that ribitol enhances cell death with JQ1, which involves a Cyt C-regulated apoptosis pathway in breast cancer cells. 

We also investigated the expression of various proteins known to play important roles in apoptosis. Ribitol and JQ1 single treatment showed little effect on the apoptosis master regulator p53 gene expression, but both had clear inhibition on anti-apoptotic protein c-Myc expression. The combination of ribitol and JQ1 increased the levels of p53 but decreased the expression of c-Myc (Figure 5B). In addition, the combined treatment also affected the expression of B-cell lymphoma 2 (Bcl-2) family proteins such as Bcl-2, Bcl-xL, and Mcl-1. Ribitol alone decreased the expression of Bcl-2 and Bcl-xL, and particularly MCL-1 expression, when compared to control and JQ1 alone (Figure 5B). JQ1 alone showed a similar effect on the expression of Bcl-2 and Bcl-xL as ribitol treatment, but less effect on Mcl-1 expression. The combined treatment of the two agents overall showed the strongest enhancement in p53 expression and a decrease in the expression of BCL-2 family proteins. 

### 3.5. Metabolic Reprogramming of Breast Cancer Cells after Ribitol and JQ1 Treatment

To assess the mechanisms involved in the observed synergy between ribitol and JQ1, we conducted a global untargeted metabolomic profile analysis to identify alterations in the metabolites of the MDA-MB-231 cells after treatment with ribitol and JQ1. The cells were treated for 3 days with 5 mM ribitol and 1 µM JQ1 and harvested for measurement of metabolites. 

#### 3.5.1. Glycolytic Suppression Dynamically Changes the Glucometabolic Profile of the MDA-MB-231 Cells 

**Figure 6:**
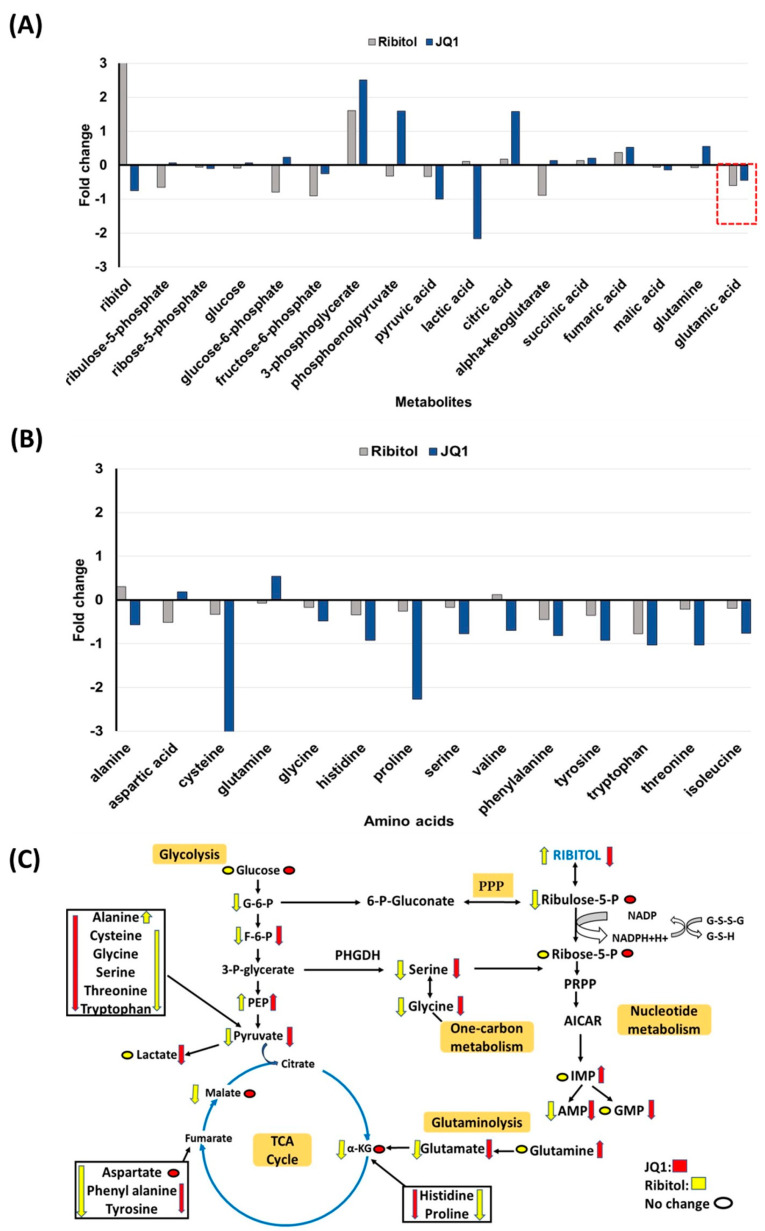
Metabolomics of MDA-MB-231 cells treated with ribitol and JQ1. (**A**) Alterations in intermediates of glycolysis and the TCA cycle. (**B**) Alteration of glucogenic and glucogenic-ketogenic metabolites and nucleotide metabolism. All glucogenic amino acid levels of tyrosine, phenylalanine and tryptophan were decreased after ribitol and JQ1 treatment compared to control. (**C**) Ribitol and JQ1 treatments decrease AMP levels and one-carbon metabolism with reduced glycine and serine levels. G-6-P: glucose-6-phosphate; F-6-P: fructose-6-phosphate: 3-P-glycerate: 3-phosphoglycerate; PEP: phosphoenolpyruvate; α-KG: α-ketoglutarate; PPP: pentose phosphate pathway; PRPP: phosphoribosyl pyrophosphate; AICAR: 5-aminoimidazole-4-carboxamide ribonucleotide; IMP: inosine 5′-monophosphate; AMP: adenosine monophosphate; GMP: guanosine monophosphate.

Interestingly, one of the most significant changes after ribitol treatment was the level of glucose 6-phosphate (G6P), a metabolic intermediate shared by both PPP and glycolysis (Figure 6). G6P levels decreased significantly in ribitol-treated samples compared to controls, and there was no significant change in the JQ1-treated group. Along with G6P, another key intermediate of the glycolysis pathway, fructose-6-phosphate (F6P) levels, also decreased significantly in the ribitol and JQ1-treated groups. Further downstream molecules of the glycolysis pathway, such as 3-phopsphoglycerate (3PG) and phosphoenol pyruvate (PEP), were increased in JQ1-treated cells compared to ribitol-treated cells. Interestingly, in agreement with our previous findings, JQ1-treated breast cancer cells showed a significant decrease in lactate levels, whereas a slight increase was detected with ribitol treatment (Figure 6). The results support the notion that inhibition of the Warburg effect is involved in JQ1-induced cell death. There was no significant change in tricarboxylic acid cycle (TCA) cycle intermediates such as α-ketoglutarate (α-KG), succinic acid, or fumaric acid (Figure 6A). Taken together, these data indicate that each agent affects glycolysis differently. Based on these data, we speculate that some breast cancer cells remain capable of using the TCA cycle to generate ATP from mitochondrial oxidative phosphorylation (OXPHOS) for survival and proliferation. 

#### 3.5.2. Changes in Gluconeogenesis, Nucleotide Synthesis and One-Carbon Metabolism after Ribitol and JQ1 Treatment 

JQ1 treatment decreases the levels of many TCA cycle intermediates and amino acids, particularly cysteine, histidine, valine, threonine, and isoleucine, with statistical significance (Figure 6B). However, there were no significant alterations in glucogenic and ketogenic amino acids in ribitol-treated cells. These findings imply that JQ1 affects mainly the TCA cycle, whereas ribitol acts on glycolysis. Glutamine levels were upregulated in the JQ1 treatment group compared to the control, but glutamate levels were downregulated in both ribitol and JQ1 treatment (red dotted line Figure 6A). These findings suggest that the influx of glutamine and glutamate into the TCA cycle is restricted, limiting the energy supply to cancer cells.

We noticed downregulated glycine and serine levels in both JQ1 and ribitol-treated cells (Figure 6C). Serine and glycine are biosynthetically linked and together provide the essential precursors for the synthesis of proteins, nucleic acids, and lipids that are crucial to cancer cells metabolic reprogramming to sustain cell growth and proliferation. Finally, limited alteration was detected in metabolites of nucleotide metabolism after ribitol and JQ1 treatment. The only significant change is the increase in inosine monophosphate (IMP) levels with JQ1 treatment, but downregulated adenosine monophosphate (AMP) levels after both JQ1 and ribitol (Figure 6C). The overall alteration in various metabolic pathways of central carbon metabolism crucial to cancer cell survival and proliferation is summarized in Figure 6C.

## 4. Discussion

Effective use of aerobic glycolysis in metabolism represents great versatility for cancer cells for survival and growth. More recent studies also demonstrate that cancer cells remain capable of utilizing oxidative phosphorylation for the production of ATP and metabolites for growth. This may well explain the difficulty in achieving therapeutic effects by applying drugs to inhibit glycolysis or oxidative phosphorylation. Furthermore, cancers are heterogeneous populations in metabolism, with a highly variable degree of efficiency in deploying each of these two major pathways for survival and proliferation. One example is breast cancer, which is currently classified into several types based on histology and marker gene expression. It is well understood that triple-negative breast cancer (TNBC) such as MBA-MB-231 has a high capacity for glycolysis, whereas ER-positive types such as MCF-7 and T-47D are oxidative phosphorylation dominant. Our early study showed that ribitol has little inhibitory effect on MCF-7 and T-47D cells [4]. However, we now show ribitol clearly inhibits the growth of MDA-MB-231 cells, and more interestingly, ribitol inhibits the migration of cells. This is in contrast to the effect of ribitol on the other two breast cancer cell lines. Perhaps more importantly, this migration inhibition is best presented when ribitol is used in combination with JQ1, clearly as a synergistic effect. This differential effect of ribitol on different types of breast cancer is important as it emphasizes that the development of metabolomic interventions must take a similar approach as the development of any combinatorial chemo- and targeting anticancer drugs. The desire to apply metabolomic intervention to a large scope of cancer types may be one of the reasons identification of this class of drugs in a clinical trial setting has been delayed. 

Epigenetic regulators are promising targets in cancer, and bromodomain and extra-terminal (BET) inhibitors such as JQ1 demonstrate anticancer effects by downregulating gene expression of oncogenic factors [24]. Preclinical evidence demonstrates the significant potential of BET inhibitors in breast cancer [25,26]. JQ1 inhibited the growth of TNBC lines in various studies [27,28,29], and the same effect was confirmed in murine xenograft models [30]. Currently, phase I/II clinical trials are exploring the safety and efficacy of BET inhibitors in solid tumors, including breast cancer and hematological malignancies [31,32,33,34,35,36]. Consistent with these findings, our current study also revealed that JQ1 alone inhibits the growth of TNBC cells. One of the main limits for the applications of JQ1 is the dose-dependent thrombocytopenia reported in clinical trials [35,36]. One approach to overcoming the toxicity and side effects is to develop better-tolerated BET inhibitors with higher clinical efficacy and toxicity thresholds. Efforts have also been made to limit the toxicity of JQ1 by combining its use with other drugs. Several studies have reported that PI3K, MEK, mTOR, and HDAC inhibitors are able to synergize with BET inhibitors in breast cancer cells [37,38,39]. This could significantly reduce the dose of BET inhibitors required to achieve efficacy. Our synergistic studies of ribitol show that the combination of ribitol and JQ1 has the highest synergism than other drug combinations. Ribitol is a metabolite normally present in small quantities in tissues and represents a new class of enhancer for the BET inhibitors. Ribitol has now been shown to have a high degree of tolerability even when administered in high doses (up to 5–10 g/kg body weight) for long-term treatment (up to at least 1 year) in both animal models and in human clinical trials (34–36). Ribitol alone has a limited inhibitory effect on triple-negative breast cancer cells, but it significantly enhances JQ1-induced cancer cell toxicity and migration inhibition. This could reduce the dose of JQ1 to achieve a therapeutic effect, potentially avoiding severe toxicity. 

Mechanisms for the synergy between ribitol and JQ1 have not yet been explored. The BET inhibitor JQ1 was developed more than 10 years ago [34,35] with the broad effect of competing with acetylation sites for histone binding to the BD of the BET protein. The binding of JQ1 replaces the BET protein in the chromatin, resulting in the dissociation of the BET protein. Changes in patterns of acetylation lead to a cascade of alterations in gene expression, benefiting many disease processes and thus serving as therapeutics. BET proteins also play important roles in tumor development by regulating the expression of multiple transcription factors, and two of them are c-Myc and Bcl-2 [32,33,34]. c-Myc is a metabolic sensor and functions as a central regulator of proliferation, differentiation, and apoptosis [35,36]. c-Myc overexpression promotes cell proliferation and accelerates the metastasis process in a variety of cancers. In our study, the ribitol and JQ1 combination decreased c-Myc expression significantly. Recent studies have linked the anticancer effect of JQ1 and other BET inhibitors to the inhibition of MYC and its downstream target genes, leading to the inhibition of key glycolytic enzymes. Our results revealed that ribitol and JQ1 treatment significantly downregulated the expression of pro-survival BCl-2 family proteins such as Bcl-2, Bcl-xL, and Mcl-1. These findings are in agreement with previous studies reporting that JQ1 inhibits cancer cell growth and promotes cell death in association with inhibition of BCL-2 and E2F1, or key transcriptional factors, including FOSL1 and androgen receptor (AR) [40]. All these factors lead to changes in the inner mitochondrial membrane that result in an opening of the mitochondrial permeability transition (MPT) pore, loss of the mitochondrial transmembrane potential, and release of cytochrome C to trigger apoptosis in cancer cells. These changes are consistent with our observation that ribitol enhances JQ1-induced cell death by apoptosis. This hypothesis is further supported by our demonstration that the combination of ribitol and JQ1 upregulated the expression of p53, a key regulator for cell survival and death that is most frequently mutated in cancer [41]. 

More recently, it has been reported that JQ1 may target multiple genes in a cell-type-dependent manner [38,39], including altering metabolism. Specifically, JQ1 inhibits the proliferation of cancer cells by restraining glycolysis, and such an effect can be partially abolished after pretreatment with 2-deoxy-D-glucose (2-DG), an inhibitor of glycolysis. JQ1 is able to restrain the glycolysis of leukemia cell lines by downregulating the rate-limiting enzymes of glycolysis, hexokinase 2, pyruvate kinase M2 (PKM2), and lactate dehydrogenase A. This effect is also mediated by the inhibitory effect of JQ1 on c-Myc, which enhances glycolysis. Our metabolomics analysis of the breast cancer cells showed that JQ1 treatment inhibits glycolysis with a significant decrease in levels of pyruvate and lactate, consistent with the early report in the leukemia cell line [42,43]. This decrease is associated with an increase in the immediate upstream metabolites 3PG and phosphoenolpyruvate. The result therefore suggests a similar mechanism involved in the JQ1-induced inhibition of cell growth and cell death in the reported cell lines. Interestingly, ribitol treatment in the cells shows an effect largely on the levels of metabolites upstream of glycolysis, with a decrease in G6P and F6P but an increase in 3PG. We therefore hypothesize that ribitol and JQ1 both act on glycolysis as inhibitors, but at different steps of the pathway. This likely creates a synergy for more efficient inhibition of glycolysis, thus more effective growth inhibition and cell death. It is worth noting that ribitol-induced alteration in the TCA cycle could also enhance the effect of altered glycolysis on the cancer cells. Both reported target cells, leukemia and MDA-MB-231, are dominant in glycolysis for their energy and metabolite production. In addition, c-Myc increases glutamine uptake by directly inducing the expression of glutamine transporters [44] and promoting the expression of the PKM2 isoform [24]. Therefore, combined treatment with ribitol and JQ1 may inhibit glutamine uptake by downregulating c-Myc expression, which further limits the energy supply essential to cancer cell survival. The upregulation of p53 by ribitol can inhibit glucose-6-phosphate dehydrogenase (G6PD), leading to an alteration in the pentose phosphate pathway (PPP). This is supported by a decrease in glucose-6-phosphate and F6P and an increase in 3-phosphoglycerate levels. However, increasing 3-phosphoglycerate does not increase serine (a precursor of nucleotide metabolism) or glycine (which participates in one-carbon metabolism) levels. This could well enhance the inhibitory effect on cancer cell survival and proliferation. Apparently, one further study will be to investigate whether this metabolic character could be a general marker for the combined JQ1 and ribitol treatment. 

## 5. Conclusions

In conclusion, our results for the first time demonstrate that a metabolite could be used in combination with a pathway-specific drug for cancer treatment with higher efficacy and reduced toxicity. As shown in the graphical abstract, we constructed a model illustrating the perturbations in energy metabolism and mechanisms of action of ribitol and JQ1. Inhibition of the c-Myc pathway leads to decreased activity of both the pyruvate kinase isoform M2 (PKM2), downregulating glycolysis, and glutamine uptake. Increasing p53 levels triggers metabolic changes and induces apoptosis by ribitol and JQ1 in breast cancer cells. Altogether, these alterations of breast cancer cell metabolism mediated by ribitol and JQ1 result in a strong induction of apoptosis and inhibition of cell growth. However, such a synergistic effect is likely restricted to a certain cancer population. Given the superiority of the combination over single drugs in our in vitro studies, further studies to understand the metabolic pathways ribitol participates in and specifically the effect of therapeutic doses of ribitol on glycolysis, the pentose phosphate pathway, and the TCA cycle are warranted.

## Data Availability

All data generated/analyzed in this study are included in this article or in the Supplementary Information Files and can be provided upon request.

## Supplementary Materials

The following supporting information can be downloaded at: https://www.mdpi.com/article/10.3390/cancers15174356/s1, Supplementary Figure S1: Synergistic effect of ribitol and JQ1 on MDA-MB-231, MCF-7, and T-47D cells cultured under both α-MEM and DMEM media. (A) Dose-dependent viability curves showing the EC_50_ for JQ1 in MCF-7, T-47D, and MDA-MB-231 cells in α-MEM media and DMEM media. (B) Synergistic plots of JQ1 and ribitol inhibited the different types of breast cancer cells, MCF-7, T-47D, and MDA-MB-231 cells, in α-MEM media and DMEM media. Supplementary Video S1: Synergistic effect of ribitol and JQ1 on MDA-MB-231 cell migration. Representative images of migration assay after the cells were treated for 48 h recorded by MuviCyte Live-Cell imaging system. File S1: Original western blots.
