# Supplementary figures and images for: Metabolic Reprogramming by Ribitol Expands the Therapeutic Window of BETi JQ1 against Breast Cancer
Source: Cancers (Basel). 2023 Sep 1;15(17):4356. doi: 10.3390/cancers15174356 (PMC10486979; doi:10.3390/cancers15174356)

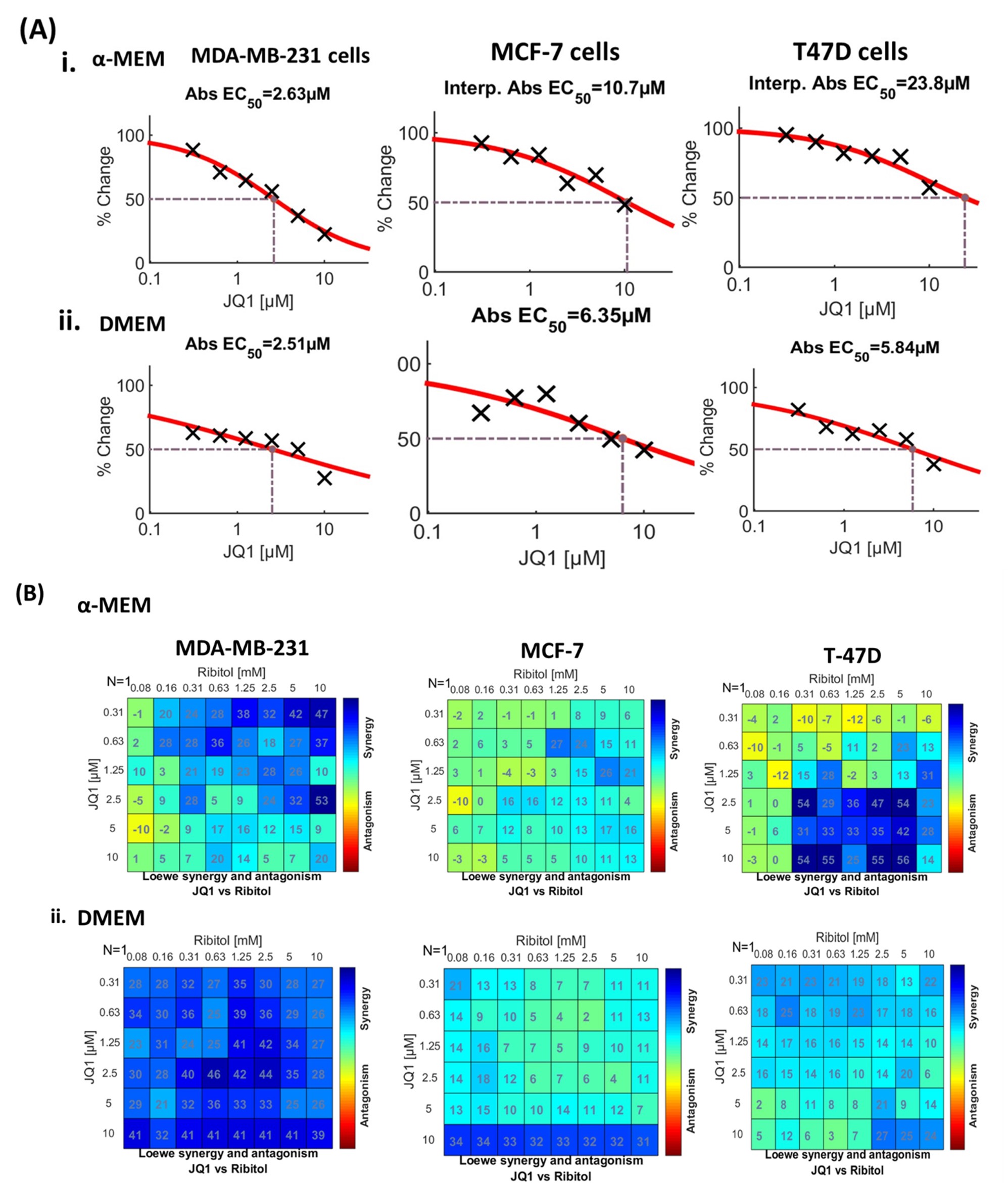

Supplement: Supplementary file 1 [file cancers-15-04356-s001.zip › cancers-2494268-Figure S1.jpg]

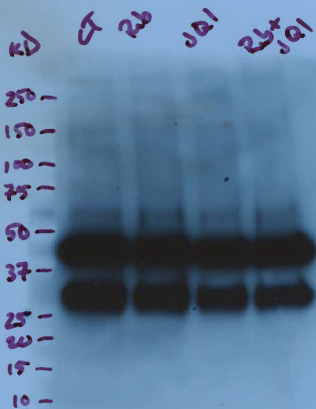

$\beta$ -Actin

# Bcl-2

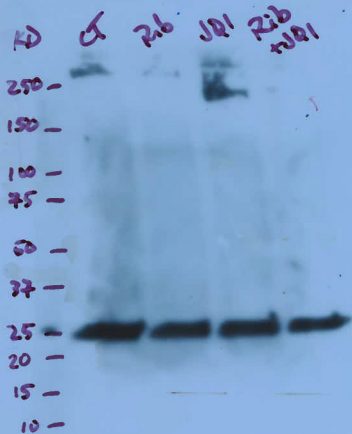

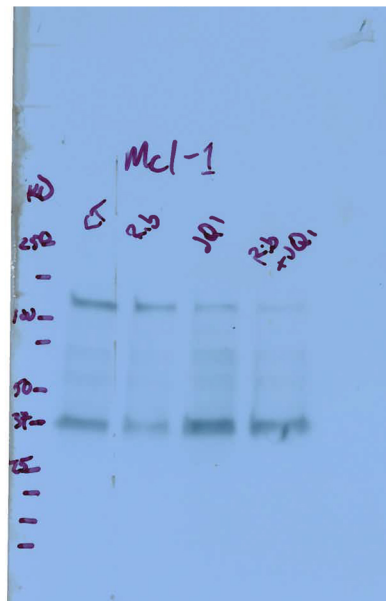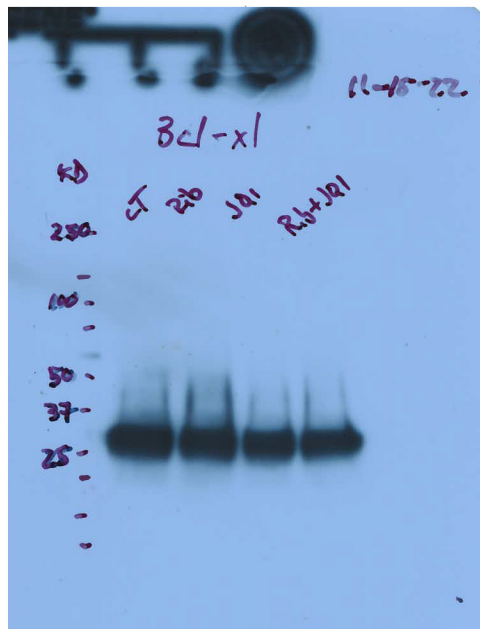

10-16-22

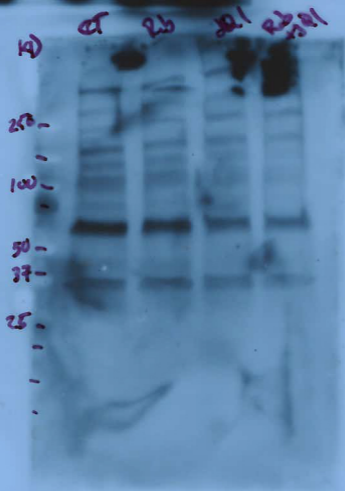

C-myc

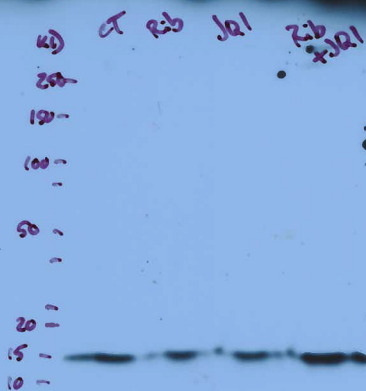

Cytochrome C

P53

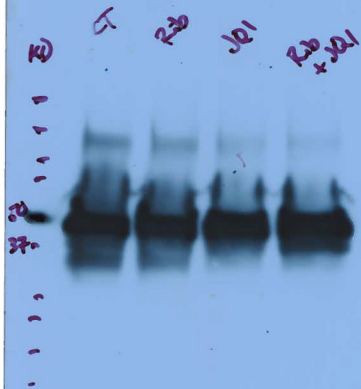

Supplement: Supplementary file 1 [file cancers-15-04356-s001.zip › cancers-2494268-File S1.pdf]
